# Efficacy of Vandetanib in Treating Locally Advanced or Metastatic Medullary Thyroid Carcinoma According to RECIST Criteria: A Systematic Review and Meta-Analysis

**DOI:** 10.3389/fendo.2018.00224

**Published:** 2018-05-03

**Authors:** Pierpaolo Trimboli, Marco Castellana, Camilla Virili, Francesco Giorgino, Luca Giovanella

**Affiliations:** ^1^Department of Nuclear Medicine and Thyroid Centre, Oncology Institute of Southern Switzerland, Bellinzona, Switzerland; ^2^Department of Emergency and Organ Transplantation, Section of Internal Medicine, Endocrinology, Andrology and Metabolic Diseases, University of Bari Aldo Moro, Bari, Italy; ^3^Department of Medico-Surgical Sciences and Biotechnologies, Sapienza University of Rome, Latina, Italy

**Keywords:** medullary thyroid carcinoma, tyrosine kinase inhibitors, vandetanib, RECIST, systematic review, meta-analysis

## Abstract

**Background:**

Vandetanib is the most largely used tyrosine kinase inhibitor (TKI) in patients with locally advanced and/or metastatic medullary thyroid cancer (MTC). Here, we conducted a systematic review on its efficacy and attempted to perform a meta-analysis adopting standardized RECIST criteria as end-points.

**Methods:**

The terms “medullary thyroid” and “protein kinase inhibitor” (then including all TKIs) were searched in PubMed, ClinicalTrials.gov, and CENTRAL. Only original studies reporting the use of Vandetanib as single agent in MTC were included. The last search was performed on October 31, 2017 and registered in PROSPERO on December 12, 2017 (n = CRD42017081537).

**Results:**

The search revealed 487 articles, and, after removing duplicates, reading title and abstract, and screening the eligible papers, 10 studied were finally included. Two papers were randomized controlled trials and eight were observational longitudinal studies. No data were available for overall survival. No heterogeneity nor publication bias were recorded in the pooled rate of complete response (0.7%) and stable disease (47%). Mild to moderate heterogeneity were recorded in the pooled rate of other endpoints. Data of the studies did not allow to perform a meta-analysis of time-to-event outcomes.

**Conclusion:**

Vandetanib should be considered as a promising treatment in advanced MTC. However, data based on RECIST endpoints do not currently provide high-level evidence on its efficacy.

## Introduction

### Rationale

Medullary thyroid carcinoma (MTC) is a thyroid tumor which can occur as hereditary (20% of cases), due to an activating germline mutation of the REarranged during Transfection (RET) protooncogene, or sporadic (80%), with a somatic RET mutation (1). While the majority of these carcinomas involves only the thyroid gland and reaches the complete cure following total thyroidectomy, a not negligible rate manifests as locally advanced and/or with distant metastases. The latter patients have a poor prognosis and need alternative treatments other than surgery. Generally, the 10-year overall survival (OS) of patients affected by MTC is 95% in patients with local disease, while this rate decreases to 75% in those with regional invasion and 40% in the presence of distant metastases (2).

### Objectives

Cytotoxic chemotherapy and external beam radiation therapy are poorly effective for metastatic MTC. For this reason, current guidelines support the use of tyrosine kinase inhibitors (TKIs) as first-line therapy in patients with significant tumor burden and symptomatic or progressive metastatic disease according to RECIST criteria (1). The pathophysiology of this type of tumor and the therapeutic approaches have been studied also by the use of animal models of MTC (3). Vandetanib is the most largely used TKI in advanced MTC, targeting the RET oncogene, the vascular endothelial growth factor receptor, and the epidermal growth factor receptor (4). Following Phase I and II trials, in 2006, the Phase III ZETA randomized clinical trial (RCT) started, based on their results, Vandetanib was eventually approved by the Food and Drug Administration (FDA) for the treatment of symptomatic or progressive MTC in patients with unresectable, locally advanced or metastatic disease in 2011 (5). Notably, TKIs are frequently associated with severe side effects, and this could be a significant limitation for their chronic use. Furthermore, the discontinuation of a TKI treatment may represent a boost for rapid increase of tumor growth and disease progression (6–8). Thus, whether to treat or not a patient with advanced MTC with TKI may be a difficult decision, and a patient-tailored approach is recommended. MTC is a rare tumor, and this limits our knowledge on the efficacy of TKI in these cancers due to the inherent difficulty in collecting adequate samples for statistical analysis.

### Research Question

Here, we have conducted a systematic review on the efficacy of Vandetanib in the treatment of locally advanced and/or metastatic MTC, attempting to perform a meta-analysis with the adoption of standardized RECIST criteria as endpoints (9, 10).

## Methods

### Study Design

The study design included studies investigating the efficacy of Vandetanib in MTC patients in terms of RECIST criteria. The present systematic review was registered on December 12, 2017 in PROSPERO (n = CRD42017081537) and performed in accordance with the Preferred Reporting Items for Systematic Reviews and Meta-Analyses (PRISMA) statement. The PRISMA checklist can be found in Table S1 in Supplementary Material.

### Participants, Interventions, and Comparators

Participants were MTC patients treated with Vandetanib due to advanced disease. There was no comparator.

### Search Strategy

To retrieve the best of published literature on the efficacy of Vandetanib in MTC, a four-step search strategy was planned. First, we identified keywords and MeSH terms in PubMed. Second, the terms “medullary thyroid” and “protein kinase inhibitor” (then including all TKIs) were searched in PubMed, ClinicalTrials.gov, and CENTRAL. Third, according to our aim, original studies reporting on the use of Vandetanib as a single agent in patients with locally advanced or metastatic MTC were included for the present analysis. Fourth, references of included studies were searched for additional papers on the same topic. The last search was performed on October 31, 2017. No language restrictions were adopted. Articles with unclear data, case reports, and series with overlapping results were excluded. Two investigators (Marco Castellana and Pierpaolo Trimboli) independently searched papers, screened titles, and abstracts of the retrieved articles, reviewed the full-texts, and selected articles for their final inclusion.

### Data Sources, Studies Sections, and Data Extraction

The same two investigators (Marco Castellana and Pierpaolo Trimboli) extracted data independently and in duplicate and in a piloted form. The following data were reported: 1. general information on the study (first author or principal investigator, year of publication, study name, study type and phase, study funding, drug in active treatment and comparator arms, duration of treatment, listed location countries, number and characteristics of patients, such as age, gender, hereditary/sporadic MTC, their previous treatments); 2. endpoints according to RECIST, including complete response (CR), partial response (PR), stable disease (SD), progressive disease (PD), objective response rate (ORR), disease control rate (DCR), progression-free survival (PFS), OS, time to response (TTR), duration of response (DOR), and PD as reason for discontinuation. Discrepancies were solved by a mutual consensus among all authors.

### Data Analysis

For CR, PR, SD, PD, ORR, DCR, and PD as reason for discontinuation, a proportion meta-analysis was performed; the number of patients with a specific endpoint was extracted from the paper or calculated from the frequency reported in the text. In addition, data on SD at 6, 12, 24, and 36 months, respectively, were searched. For time-to-event outcomes (OS, PFS, TTR, and DOR), a log-rank analysis was attempted. For statistical pooling of the data, the fixed method was used. Pooled data are presented with 95% confidence intervals (95% CI) and displayed using a Forest plot, when possible. *I*_2_ index was used to quantify the heterogeneity among the studies, a significant heterogeneity being defined as an *I*_2_ value >50%. Egger’s test was also carried out to evaluate the presence of significant publication bias, and a *p* value <0.05 identified the presence of bias. Statistical analyses were performed using the StatsDirect statistical software version (StatsDirect Ltd., Altrincham, UK).

## Results

### Study Selection and Characteristics

**Figure 1:**
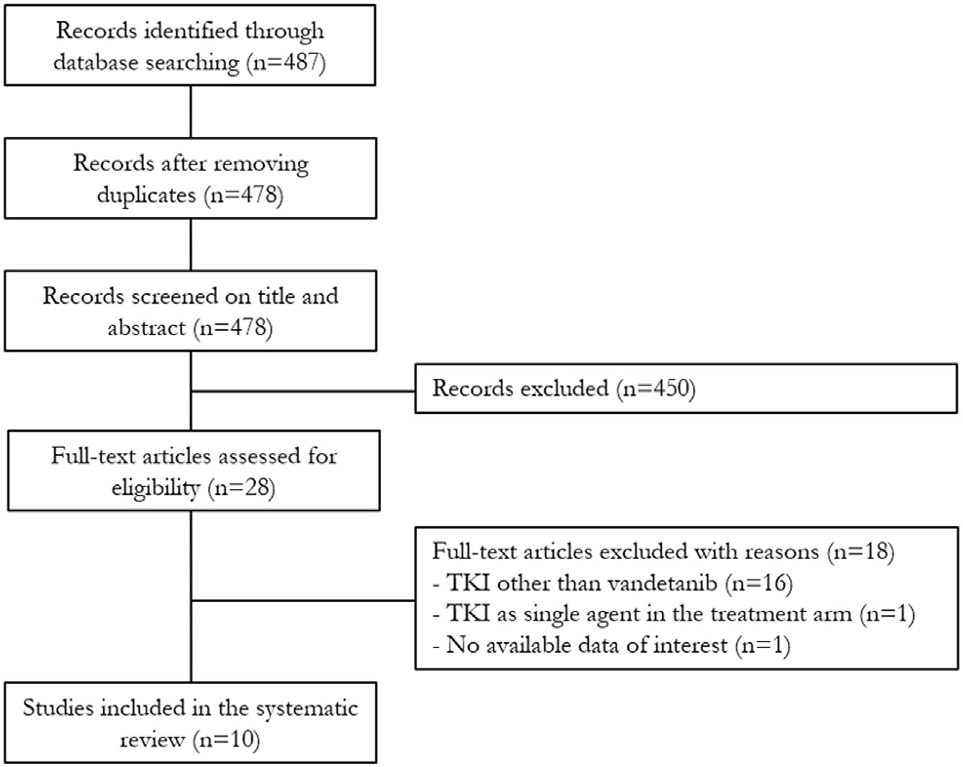
Flowchart of the study selection process.

The search revealed 487 potentially relevant articles. After duplicates removal, 478 were initially selected, and by reading their title and abstract 450 studies were excluded. The full-text of the remaining 28 papers was obtained and evaluated; 18 articles were excluded because of several reasons (i.e., the patients received a TKI other than Vandetanib, Vandetanib was associated with a proteasome inhibitor in the active treatment arm, or no data useful for the meta-analysis were reported). Finally, 10 studies were included in the present systematic review (11–20) (Figure 1).

### Synthesized Analysis

The characteristics of the included articles are summarized in Table 1. The studies were published between 2010 and 2017, and reported cohorts of 14–331 MTC patients. Two studies were RCTs, eight were observational longitudinal studies (OLSs); five studies were Phase II OLSs, single-country and single-arm. Five studies were sponsored by industry. Participants were outpatients diagnosed with histologically confirmed, unresectable, locally advanced, or metastatic MTC. Three studies included children and adolescents. A total of 496 patients were treated with Vandetanib in these 10 studies; 100 patients were given placebo in only 1 RCT. One RCT compared the effects of two doses of Vandetanib, i.e., 150 and 300 mg/day. The mean age was 51.2 ± 6.8 years, 56.1% of the patients were male, 24.8% were hereditary MTC, 95.5% had distant metastases. The median duration of treatment ranged from 8.7 to 39.2 months. Among Vandetanib-treated patients, the median PFS ranged from 7 to 30.5 months (finding from five studies), TTR from 4.3 to 18.6 months (three studies), and DOR from 10.2 to 22.2 months (three studies). Among placebo-treated, a PFS of 19.3 and a DOR of 16.3 months were reported [in one study (13)].

**Table 1:** Characteristics of included studies.

Reference	Study type	Identifier of trial	Vandetanib dose (mg/day)	Duration of treatment (months, median, and ranges)	Comparator	MTC patients (active/comparator)	Countries	Age of MTC patients (years)	Phase	Industry sponsor
Robinson et al. (11)	OLS	NCT00358956	100–300	8.7 (0.1–16.7)	–	19/–	8 countries	45 (median)	II	AstraZeneca
Wells et al. (12)	OLS	NCT00098345	300	–	–	30/–	2 countries	49 (median)	II	Sanofi
Wells et al. (13)	RCT	NCT00410761 (ZETA)	300	22.5 (NR)	Placebo	231/100	23 countries	52 (mean)	III	AstraZeneca
Fox et al. (14)	OLS	–	70–150^a^	25.2 (1.8–48.5)	–	16/–	USA	14 (mean)	I/II	–
Chougnet et al. (15)	OLS	–	50–300	9.7 (0.3–36.0)	–	60/–	France	58 (mean)	IV	–
Akshintala et al. (16)	OLS	NCT00514046	100^a^	39.2 (1.8–78.4)	–	17/–	USA	–	II	–
No author listed, 2016 (17)	RCT	NCT01496313	150–300	–	–	81/–	9 countries	52 (mean)	IV	Sanofi
Tiedje et al. (18)	OLS	–	–	–	–	10/–	Germany	58 (mean)	IV	–
Uchino et al. (19)	OLS	NCT01661179	100–300	14.1 (0.2–21.5)	–	14/–	Japan	52.5 (median)	I/II	AstraZeneca
Werner et al. (20)	OLS	–	300	–	–	18/–	Germany	48 (median)	IV	–

*^a^Dose is reported as mg/m^2^/day*.

*MTC, medullary thyroid carcinoma; OLS, observational longitudinal study; RCT, randomized controlled trial*.

**Figure 2:**
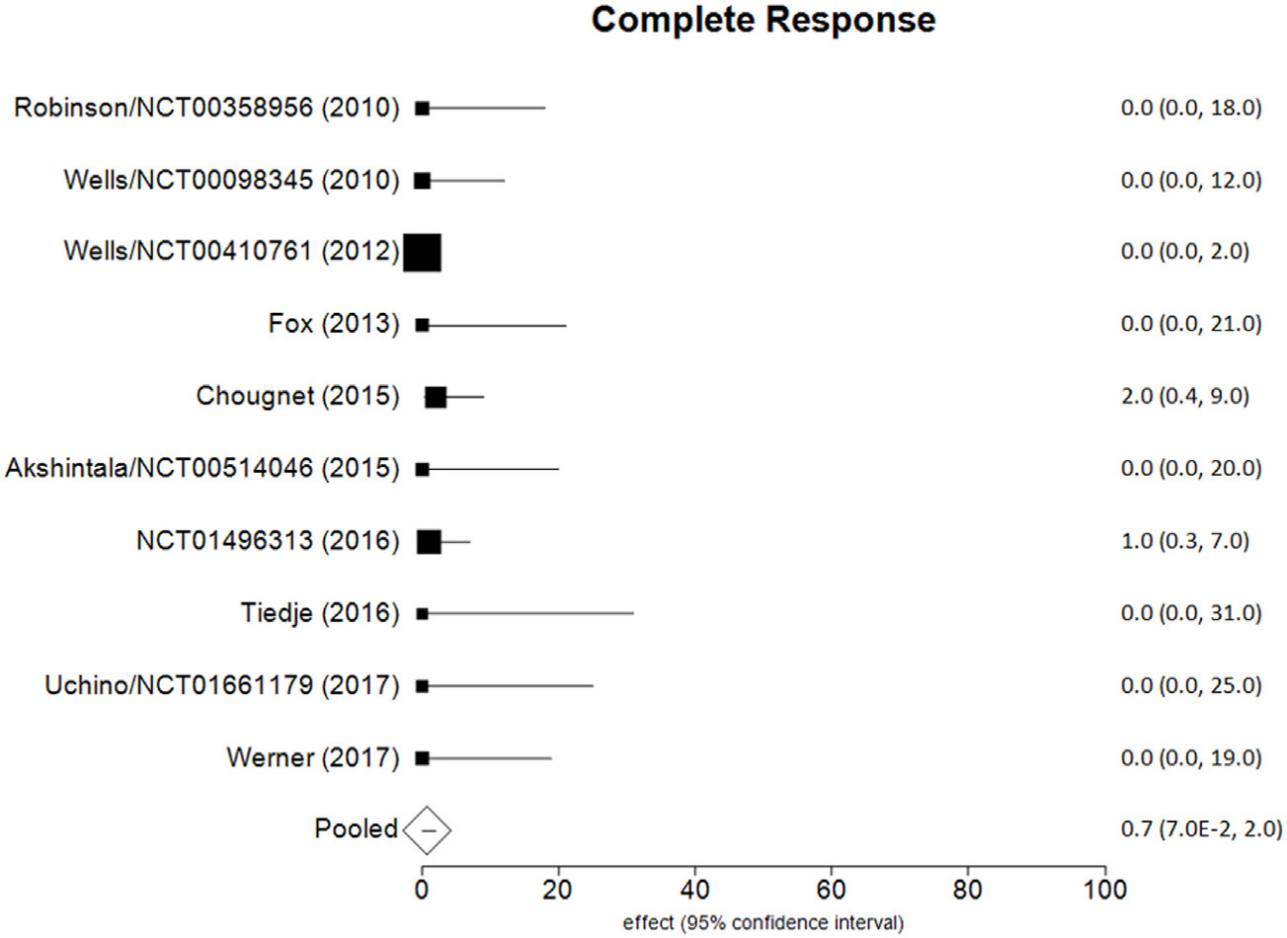
Forest plot of data on complete response (fixed effect).

**Figure 3:**
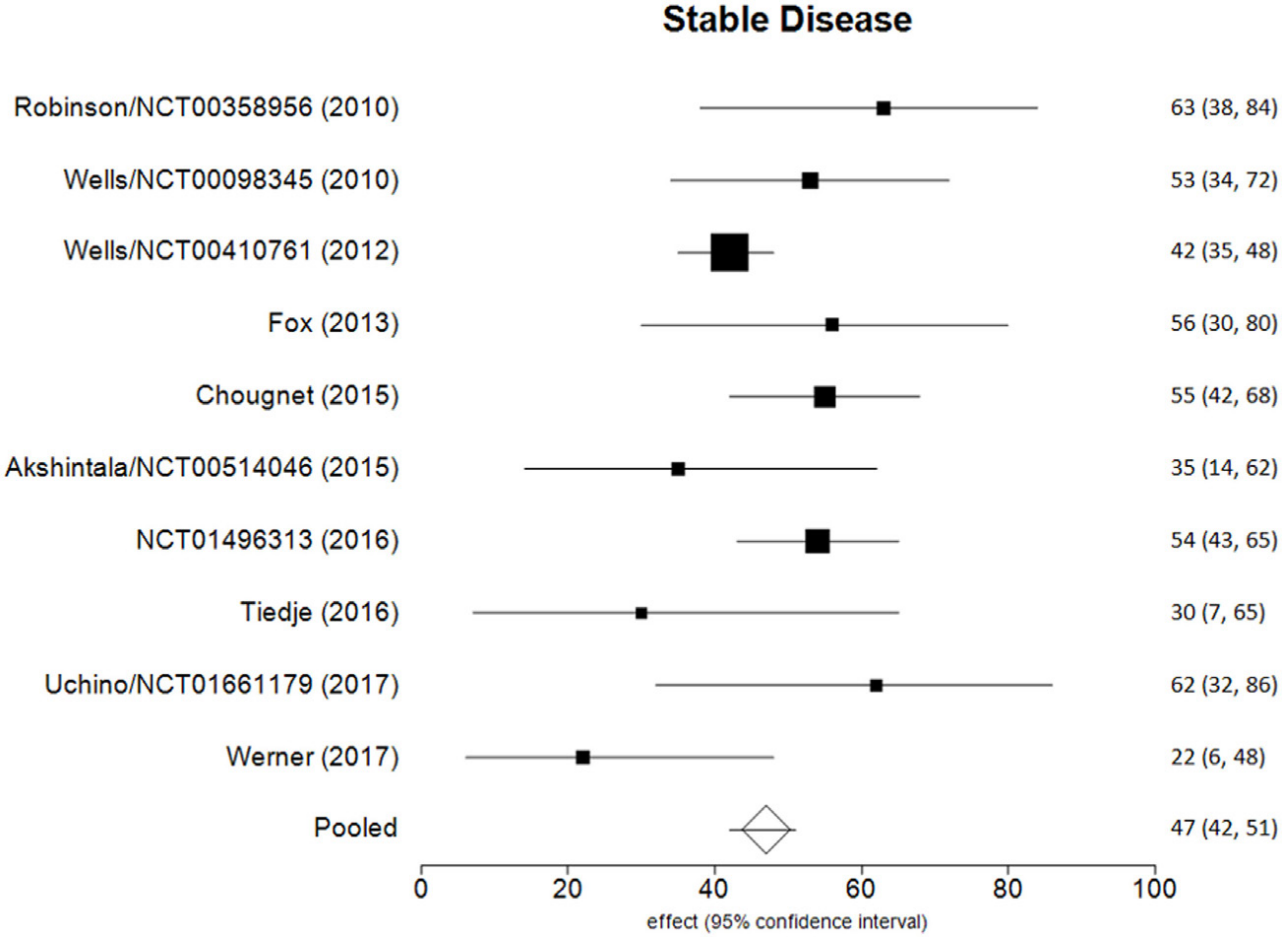
Forest plot of data on stable disease (fixed effect).

Data of RECIST endpoints available for the meta-analyses are detailed in Table 2. Remarkably, no data were available on OS; among other endpoints, CR, PR, SD, PD, ORR, and DCR were reported in all included studies, while PD as reason for discontinuation in six of them. No heterogeneity nor publication bias were recorded in the pooled rate of CR (0.7%) and SD (47%) (Figures 2 and 3). Data on the rate of SD during the study (i.e., at 6, 12, or 24 months) were reported in few studies, and thus we were not able to perform a meta-analysis on this endpoint. No publication bias and mild-to-moderate heterogeneity were recorded in the pooled rate of the other end-points (Table 3). Data on the included studies did not allow us to perform a meta-analysis on time-to-event outcomes (PFS, TTR, and DOR) because only one study included a placebo arm (13).

**Table 2:** Data available in the included studies and extracted from the meta-analysis.

Reference	PFS	OS	CR	PR	SD	PD	ORR	DCR	TTR	DOR	PD as reason for discontinuation of Vandetanib
Robinson et al. (11)	Yes	No	Yes	Yes	Yes	Yes	Yes	Yes	No	No	Yes
Wells et al. (12)	Yes	No	Yes	Yes	Yes	Yes	Yes	Yes	Yes	Yes	Yes
Wells et al. (13)	Yes	No	Yes	Yes	Yes	Yes	Yes	Yes	No	Yes	Yes
Fox et al. (14)	No	No	Yes	Yes	Yes	Yes	Yes	Yes	No	No	Yes
Chougnet et al. (15)	Yes	No	Yes	Yes	Yes	Yes	Yes	Yes	No	No	Yes
Akshintala et al. (16)	No	No	Yes	Yes	Yes	Yes	Yes	Yes	Yes	No	No
No author listed, 2016 (17)	No	No	Yes	Yes	Yes	Yes	Yes	Yes	Yes	Yes	No
Tiedje et al. (18)	Yes	No	Yes	Yes	Yes	Yes	Yes	Yes	No	No	No
Uchino et al. (19)	No	No	Yes	Yes	Yes	Yes	Yes	Yes	No	No	Yes
Werner et al. (20)	No	No	Yes	Yes	Yes	Yes	Yes	Yes	No	No	No

*CR, complete response; DCR, disease control rate; DOR, duration of response; ORR, objective response rate; OS, overall survival; PD, progressive disease; PFS, progression-free survival; PR, partial response; SD, stable disease; TTR, time to response*.

**Table 3:** Results of meta-analysis of RECIST endpoints.

	Pooled rate [95% confidence intervals (95% CI)]	*I*_2_ index (95% CI)	Egger’s test (95% CI)	*p*
Complete response	0.07% (0.007–0.2)	0% (0–53)	0.14 (−0.15 to 0.44)	0.296
Stable disease	47% (42–51)	45% (0–72)	0.47 (−1.69 to 2.63)	0.628
Objective response rate	35% (31–40)	71% (33–83)	−0.51 (−3.52 to 2.48)	0.701
Disease control rate	83% (80–86)	69% (27–82)	−0.98 (−3.66 to 1.70)	0.422
Partial response	35% (31–40)	73% (40–84)	0.33 (0.24–0.43)	0.755
Progressive disease (PD)	14% (11–17)	61% (1–79)	0.59 (−1.84 to 3.01)	0.593
PD as reason for discontinuation of Vandetanib	30% (26–35)	65% (0–83)	−0.7 (−4.91 to 3.50)	0.685

*I_2_ index quantified the heterogeneity among the studies, and a value >50% indicated significant heterogeneity; Egger’s test evaluated the publication bias, identified by a p value <0.05*.

## Discussion

### Summary of Findings

The aim of the current review was to identify the best available evidence on the efficacy of Vandetanib in treating locally advanced or metastatic MTC. Indeed, Vandetanib is currently the most largely used TKI for advanced MTC. To the best of our knowledge, this is the first systematic review and meta-analysis on this topic focusing on the RECIST criteria as benchmark. Notably, most of the retrieved papers (both original reports and reviews) identified by our search strategy focused on the side-effects, rather than on the efficacy of TKI, and only two RCTs and eight OLSs were eligible for inclusion in our analysis. As a major result, no analysis could be performed for OS because this finding was not reported in any of these 10 articles. The pooled CR and SD were the most statistically relevant results; less than 1 in 100 MTC patients had a CR to Vandetanib and about a half (47%) showed a SD at the end of the study. The other dichotomous endpoints presented statistical limitations (i.e., significant heterogeneity), while time-to-event outcomes could not be meta-analyzed due to the design of the studies.

As mentioned above, OS of MTC patients is significantly reduced in the presence of metastases (2). Besides the local effects caused by metastases in the liver, bone, lung, and mediastinal region, patients with advanced MTC may suffer from diarrhea (due to hypercalcitoninemia) or from Cushing’s syndrome due to ectopic ACTH or CRH secretion (1). Remarkably, TKI treatment might cause side effects, diminishing the patient’s quality of life. The more common adverse effects are gastrointestinal (diarrhea, nausea, and decreased appetite), cardiovascular (hypertension, QTc prolongation), dermatological (acne, skin rash), and general symptoms such as fatigue (6). Adverse events may occur in mild or severe forms; dose reduction may lead to improvement of side effects in mild cases while in severe forms Vandetanib should be interrupted until symptoms improve (7). Since metastatic MTC is incurable, it is necessary to consider that usually TKI treatment must be continued chronically, and the above side effects may impact on the patients’ quality of life (6). Furthermore, the long-term effects of the chronic use of Vandetanib are not known (1). Therefore, once metastases have appeared and/or there is a local cervical invasion, the clinician must carefully identify the best treatment considering the possibility to slow tumor progression but also the risk of side effects in the context of the best quality of life possible (8). On this ground, recent studies are proposing alternative treatments that could lead to a more individually tailored treatment (21).

A meta-analysis on TKIs for advanced metastatic thyroid cancer, including several cancer types, has been recently published. Six RCTs were included, one of which herein included also (13). Higher PFS was reported among Vandetanib- and Cabozantinib-treated patients with respect to controls (HR = 0.36; 95% CI, 0.22–0.58) (22). However, since these authors included in their meta-analysis studies with different therapies, their results could not be compared with those in our meta-analysis.

### Limitations

Some potential limitations of our meta-analysis should be addressed. According to our search strategy, we could find both RCTs and OLSs. The study design of these papers was obviously highly different (duration of the study, dose of Vandetanib used) and the series included different patient types (i.e., adults/adolescents, patients previously treated with systemic therapy or not, hereditary/sporadic MTC). Also, some studies adopted RECIST 1.0 (9) to evaluate the response to therapy, while others used the version 1.1 (10). All of these discrepancies might affect the herein reported pooled results. On February 2018, then after our literature search, an update of NCT00514046 was published by Kraft et al. (23); endpoints according to RECIST did not differ from the one reported by Akshintala et al. (16).

### Conclusion

This study demonstrates that a negligible rate of patients with advanced MTC treated by Vandetanib has a CR at the end of the study and less than a half has SD. Vandetanib, the most largely used therapy in advanced MTC, should be considered as a promising treatment in these patients. However, data on RECIST endpoints to be analyzed are limited, and the overall evidence on the efficacy of Vandetanib in advanced MTC should thus be considered as low at present. Furthermore, RCTs are needed to obtain more robust information, especially in terms of OS.

## Ethics Statement

This article does not contain any studies with human participants or animals performed by any of the authors.

## Author’s Note

This systematic review was registered in PROSPERO (n = CRD42017081537).

## Author Contributions

PT, MC, and LG conceived and designed the study. PT, MC, and CV performed the literature search; PT analyzed the data; FG and LG critically reviewed the results. All the authors contributed to write the manuscript.

## Conflict of Interest Statement

The authors declare that the research was conducted in the absence of any commercial or financial relationships that could be construed as a potential conflict of interest.
